# Role of imaging in the applicability of irreversible electroporation for the management of pancreatic adenocarcinoma

**DOI:** 10.1590/0100-3984.2022.0032-en

**Published:** 2023

**Authors:** Thiago Pereira Fernandes da Silva, Raquel Andrade Moreno, Rodrigo Pamplona Polizio, Rayssa Araruna Bezerra de Melo, Antônio Luiz de Vasconcelos Macedo, Luiz Tenório Siqueira de Brito

**Affiliations:** 1 Hospital Vila Nova Star, Rede D’Or, São Paulo, SP, Brazil.

**Keywords:** Electroporation/methods, Carcinoma, pancreatic ductal/pathology, Pancreatic neoplasms/pathology, Tomography, X-ray computed, Magnetic resonance imaging, Eletroporação/métodos, Carcinoma ductal pancreático/patologia, Neoplasias pancreáticas/patologia, Tomografia computadorizada, Ressonância magnética

## Abstract

Pancreatic ductal adenocarcinoma is one of the most aggressive malignant neoplasms, with a one-year survival rate below 20%. Axial methods (computed tomography and magnetic resonance imaging) play a fundamental role in the diagnosis and staging of the disease, because they provide adequate anatomical resolution in the assessment of key structures, mainly vascular structures. Pancreatic ductal adenocarcinoma is most often discovered in advanced stages, when surgical resection is no longer feasible. In that scenario, minimally invasive treatment alternatives have been developed in attempts to change the natural history of the disease. Irreversible electroporation, an interventional procedure that minimizes deleterious effects on adjacent tissues, has proven useful for the treatment of tumors traditionally considered unresectable. Despite the growing acknowledgment of this technique as a tool for the management of pancreatic ductal adenocarcinoma, it is still relatively unknown among radiologists. In this study, we sought to provide an overview of the main characteristics and eligibility criteria that must be considered for the indication of irreversible electroporation in cases of pancreatic ductal adenocarcinoma.

## Abstract (pt)

O adenocarcinoma ductal de pâncreas é uma das neoplasias malignas mais agressivas, com taxas de sobrevivência anuais inferiores a 20%. Os métodos axiais (tomografia computadorizada e ressonância magnética) têm papel fundamental no diagnóstico e estadiamento da doença, por fornecerem adequada resolução anatômica na avaliação de estruturas-chave, principalmente vasculares. O adenocarcinoma ductal de pâncreas é frequentemente descoberto em estágios avançados e sem viabilidade de ressecção cirúrgica, e nesse cenário o desenvolvimento de alternativas terapêuticas minimamente invasivas tem sido ainda mais importante para a mudança de sua história natural. A eletroporação irreversível, procedimento intervencionista que minimiza efeitos deletérios nos tecidos adjacentes, vem se destacando no tratamento de lesões tradicionalmente consideradas irressecáveis. Essa técnica, apesar de ganhar cada vez mais espaço no manejo terapêutico do adenocarcinoma ductal de pâncreas, ainda é pouco familiar aos radiologistas. Neste estudo, buscamos expor, de forma sucinta e didática, os fundamentos da técnica, as principais características de imagem e os critérios de elegibilidade que devem ser considerados para indicação da eletroporação irreversível nessa doença.

## INTRODUCTION

Pancreatic ductal adenocarcinoma (PDAC) accounts for 95% of malignant pancreatic tumors and is the third leading cause of cancer death in Western countries. The five-year survival rate is 4%, the lowest among gastrointestinal neoplasms. The main determinant of this unfavorable prognosis is the indolent onset of the disease, which is oligosymptomatic, making early diagnosis difficult^(1)^.

**Figure 1:**
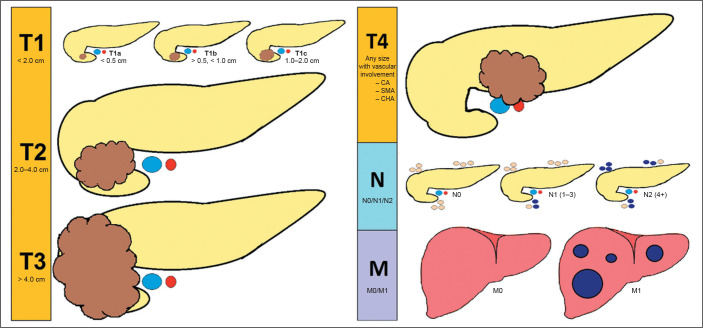
Representation of the American Joint Committee on Cancer international system of classification of tumors^(2)^, based on imaging assessment of the tumor (T), lymph nodes (N), and metastases (M). (CA, celiac artery; SMA, superior mesenteric artery; CHA, common hepatic artery).

The therapeutic management of PDAC requires a multidisciplinary team, which should mainly include oncologists, surgeons, radiotherapists, and interventional radiologists. Staging (a critical step) follows the American Joint Committee on Cancer international system of classification of tumors^(2)^ (Figure 1), which classifies imaging findings into stages with prognostic relevance (Table 1). Its criteria consider the dimensions of the tumor and the presence of lymphadenopathy/metastases. In addition, the National Comprehensive Cancer Network guidelines for the clinical and surgical management of PDAC establish criteria for resectability on a case-by-case basis, depending on the anatomical relationships between the tumor and its surroundings (Table 2).

**Table 1:** Staging of PDAC, based on the tumor-node-metastasis system outlined in the Cancer Staging Manual of the American Joint Committee on Cancer, 8th edition^(2)^.

Category	PDAC stage						
	IA	IB	IIA	IIB	III	III	IV
T	T1	T2	T3	T1-T3	T4	T1-T4	T1-T4
N	N0	N0	N0	N1	N0-N2	N2	N0-N2
M	M0	M0	M0	M0	M0	M0	M1

T, tumor; N, (lymph) node; M, metastasis.

**Table 2:** Surgical classification and resectability criteria for pancreatic cancer^(1)^.

	Vascular structures		Resectable	Borderline resectable	Unresectable
Vascular structures	Venous	Portal vein or superior mesenteric vein	No contact Contact < 180° without contour irregularities	Contact > 180° Contact < 180° with deformity/ thrombosis, not precluding resection or reconstruction Contact with the inferior vena cava	Involvement precluding resection or reconstruction Contact with proximal jejunal drainage branches
	Arterial	Common hepatic artery	No contact	Contact, without extension to the celiac artery or the hepatic bifurcation	Extension to the celiac artery/ hepatic bifurcation
		Celiac artery	No contact	No contact (head) Contact < 180° (body/tail)	Contact > 180°^f^
		Superior mesenteric artery	No contact	Contact < 180°	Contact > 180° Contact with the first jejunal branch of the superior mesenteric artery
		Aorta	No contact	No contact	Any contact
		Anatomical variants^*^	None	Right accessory hepatic artery or artery of aberrant origin (variable degree of contact)^*^	-
Other structures		-	-	-	Metastasis (including distal lymph nodes)

* A tumor in contact with arterial anatomical variants may be considered borderline resectable depending on the experience of the surgeon(s).

† As an exception, the National Comprehensive Cancer Network allows a tumor in contact with the celiac artery (> 180°) to be classified as borderline resectable; provided that the aorta and gastroduodenal artery remain patent and candidates for vascular reconstruction^(1)^.

Up to 90% of patients with PDAC have advanced (stage III) or metastatic (stage IV) tumors at diagnosis^(1)^, which makes a surgical approach less feasible. In this context, therapeutic options have been sought, culminating in the development of minimally invasive methods in the last decade, including those employing ionizing radiation (percutaneous stereotactic radiotherapy) or high-frequency sound energy (high-intensity ultrasound), as well as thermal methods, such as radiofrequency ablation (by microwave emission) and cryoablation^(3,4)^, all of which complement traditional chemoradiotherapy regimens. A new non-thermal ablation technique, known as irreversible electroporation (IRE), has recently been introduced. It has the characteristic of treating a tumor without changing the tissue temperature, thus providing greater safety in cases of PDAC by preserving the nerves, bowel loops, and vasculature in proximity to the index tumor.

## IRE: FUNDAMENTAL CONCEPTS

**Figure 2:**
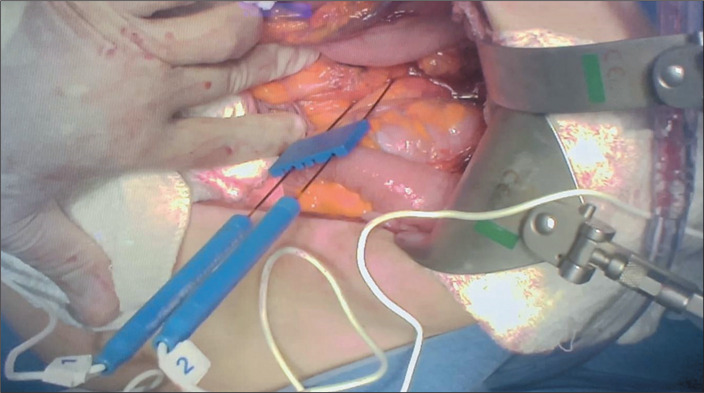
Intraoperative IRE.

In IRE, electrodes are inserted around a tumor to generate an electric field (Figure 2). Multiple cycles of short, high-voltage electrical pulses (1.5-3.0 kV) are generated in the ablation zone, altering the transmembrane potential of tumor cells and generating “pores” in the lipid bilayer of cell membranes, thus increasing their permeability. With the application of increasingly higher currents, this transitory alteration becomes irreversible, leading to a loss of homeostasis and tumor cell death by apoptosis^(5)^. In this process, the so-called “vascular block” coexists, where there is almost complete cessation of blood flow to the ablation site, due to direct vasoconstriction (resulting from electrical stimulation of the smooth muscles) and indirect vasoconstriction (mediated by the sympathetic nervous system).

The physical characteristics of IRE make it particularly attractive for use in the treatment of locally advanced PDAC, given that the use of targeted electrical energy damages the tumor cell membranes while sparing extracellular macromolecules and connective tissue, thus preserving delicate adjacent structures, such as the bile ducts, bowel loops, and vascular walls^(6)^. Because it does not generate heat, IRE avoids the heat-sink effect, a phenomenon related to the presence of large-caliber vessels near the ablation zone, which reduces the effectiveness of thermal ablation methods^(7)^. It can be performed percutaneously, guided by computed tomography (CT) or ultrasound, or intraoperatively, at the surgical site (immediately after resection of the main tumor), with the aid of ultrasound for proper positioning of the electrodes^(7)^. Currently, there is only one commercially available, IRE-specific electrode kit (NanoKnife System; AngioDynamics Inc., Latham, NY, USA).

It is necessary to be aware of the eligibility criteria for IRE. The procedure requires general anesthesia and deep neuromuscular block. Because of the effects (mainly cardiac effects) that electrical stimulation has on blood vessels and muscle tissues, there are cardiovascular conditions that contraindicate it, such as a history of ventricular arrhythmias, pacemakers, poorly controlled hypertension, and decompensated congestive heart failure. A history of epilepsy is an absolute contraindication. In addition, the procedure is avoided when there is evidence of clear involvement of the walls of hollow viscera, such as the duodenum and stomach, because of the high risk of rupture. Attention should also be given to patients with obstruction of the bile ducts or the portal vein, and it is necessary to treat these complications before performing the IRE procedure, either with the interposition of a biliary prosthesis or biliary-enteric anastomosis, in the first case, or the introduction of a portosystemic stent, in the second, given the risk of stenosis or occlusion of these delicate structures due to the edema resulting from the procedure^(8)^.

## IRE APPLICATIONS

The main indication for IRE is for the treatment of tumors classified as stage III, which is the stage at which the greatest proportion of PDACs are diagnosed. Most stage III PDACs are considered unresectable or borderline resectable. However, advances in surgical technique and preoperative/intraoperative treatment have made it possible to perform surgical resection with tumor-free margins, even in this unfavorable scenario. Fromer et al.^(9)^ gathered evidence that challenged traditional conventions in locally advanced disease and proposed subclassifying PDAC according to the site of vascular involvement (Table 3), with the aim of expanding the spectrum of patients eligible for surgery. In this context, we propose that IRE become part of the therapeutic armamentarium, either intraoperatively, increasing the chance of achieving tumor-free margins, or as a stand-alone procedure, in patients who are not candidates for a surgical approach, with the objective of achieving local control and of having a positive impact on survival and morbidity, as it has recently been shown to do^(10)^. It should be borne in mind that such practices, in their consolidation phase, should be considered on a case-by-case basis and are applicable only at specialized centers with experience in the multidisciplinary treatment of the disease.

**Table 3:** Proposal by Fromer et al.^(9)^ for the classification of locally advanced (stage III) PDAC, analyzed together with the application of IRE proposed in the present study for management of the disease.

Parameter	Classification system proposed by Fromer et al.^(9)^				Proposed application of IRE	
	> 180° arterial involvement	Subclassi­fication	Resectability		Aplicability	Objective
With or without venous involvement (of the portal vein or superior mesenteric vein) that does not preclude reconstruction	Celiac artery or common hepatic artery	IIIa	Possible (modified Appleby procedure)^*^	⇒	Intraoperative	Resection with tumor-free margins
	Superior mesenteric artery	IIIb1	Low^*^	⇒	Intraoperative or stand-alone	Resection with tumor-free margins Local control; positive impact on morbidity and mortality
	Celiac artery + superior mesenteric artery	IIIb2	Very low (selected cases)^†^			
With venous involvement (of the portal vein or superior mesenteric vein) that precludes reconstruction	No arterial involvement	IIIc1	Not viable^§^	⇒	Stand-alone	Local control; positive impact on morbidity and mortality
	Celiac artery	IIIc2				
	Superior mesenteric artery	IIIc3				
	Celiac artery + superior mesenteric artery	IIIc4				

* En bloc resection of the celiac artery with anastomosis between the common hepatic artery and the gastroduodenal artery, to maintain hepatic and gastroduodenal flow. Commonly, there is resection of the distal pancreas and spleen.

† Arterial reconstruction is mandatory when there is involvement of the superior mesenteric artery, in order to allow adequate irrigation of the intestines; this makes the procedure considerably more complex (and increases postoperative morbidity) than those involving the celiac artery, common hepatic artery, or both.

‡ The involvement of these two large visceral arterial trunks makes the procedure very complex. Resection of Stage IIIb2 tumors should be considered only after extensive patient counseling and assessment of the potential risks and benefits.

§ The surgical complexity represented by involvement of multiple vessels makes resection unfeasible in these situations, except under circumstances of scientific/experimental investigation.

## IMAGING CRITERIA FOR THE INDICATION OF IRE

### Anatomy and protocols for the initial assessment of PDAC

**Figure 3:**
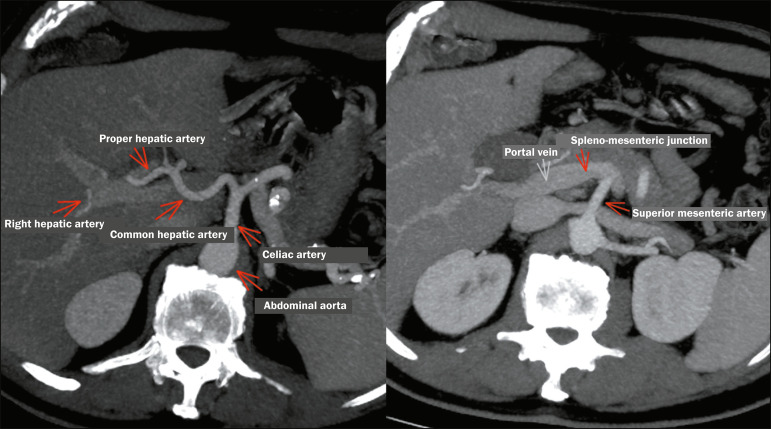
Vascular anatomy relevant to pancreatic adenocarcinoma.

High-resolution CT is the main method employed for the diagnostic staging of pancreatic cancer. Figure 3 identifies the vessels that are the most relevant in the staging of PDAC. In selected cases, magnetic resonance imaging can be an alternative or a complement to CT, such as those in which iodinated contrast is contraindicated and those in which it is necessary to investigate questionable findings (e.g., liver nodules).

### Arterial involvement

**Figure 4:**
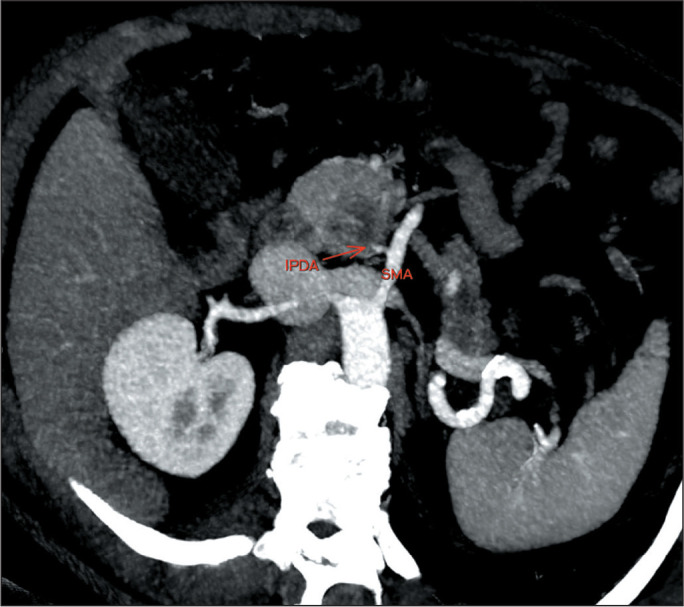
Axial maximum intensity projection reconstruction of a contrast-enhanced CT scan of the abdomen, showing a pancreatic tumor in a 61-year-old man. There is contact (< 180°) between the tumor and the superior mesenteric artery (SMA), with amputation of the inferior pancreaticoduodenal artery (IPDA, first branch of the SMA). Borderline-resectable tumor. IRE was used as an adjuvant intraoperative technique.

**Figure 5:**
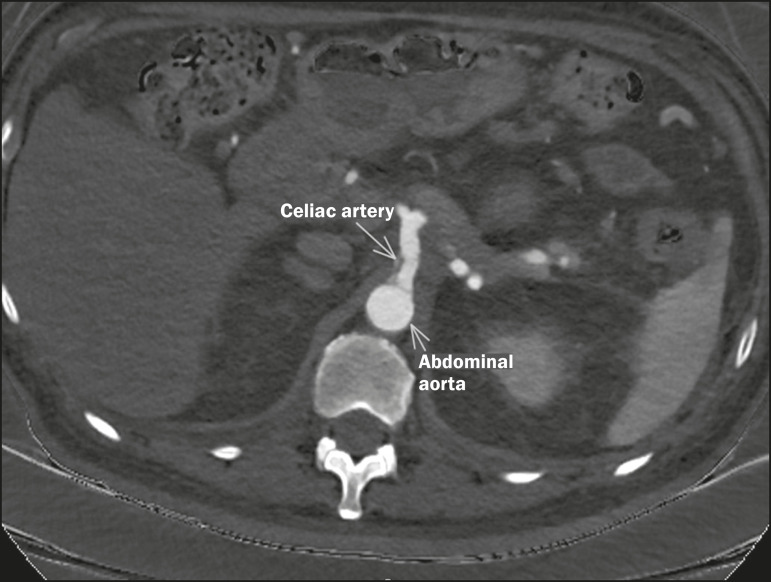
Axial contrast-enhanced CT scan of the abdomen, in the arterial phase, showing a tumor in the pancreatic body in a 78-year-old man. A comparison between the imaging aspects of the acquisition with a dual-energy protocol depicting the tumor in contact (> 180°) with the celiac artery and in marginal contact with the abdominal aorta. Unresectable tumor. The dual-energy technique increases the contrast between different tissues by processing a set of acquisitions at different voltages. Benefits over the conventional technique include better differentiation between the tumor and healthy pancreatic parenchyma, optimized vascular assessment, reduced tomographic beam attenuation artifacts caused by metallic materials (e.g., surgical clips and biliary stents), and improved image quality when the acquisition is suboptimal (e.g., reduced renal or cardiac function, which alter the circulation dynamics of iodinated contrast in the bloodstream). IRE was performed as a stand-alone procedure.

**Figure 6:**
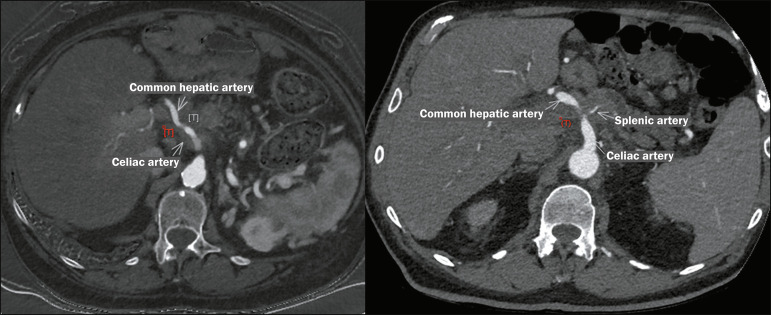
Axial contrast-enhanced CT scan of the abdomen, in a maximum intensity projection, showing a tumor (T) in the pancreatic body in a 69-year-old woman, in contact (> 180°) with the celiac artery and the common hepatic artery. Unresectable tumor. Involvement of the splenic artery, with caliber reduction, and splenic vein thrombosis are also observed. IRE was used as a stand-alone procedure, in combination with chemoradiotherapy.

As illustrated in Figures 4, 5, and 6, arterial involvement is the main determinant of resectability in cases of PDAC. The cases depicted were classified as eligible for IRE after the staging criteria had been analyzed. Unresectable tumors were treated with CT-guided percutaneous IRE, whereas borderline-resectable tumors were resected and treated with intraoperative IRE.

### Venous involvement

**Figure 7:**
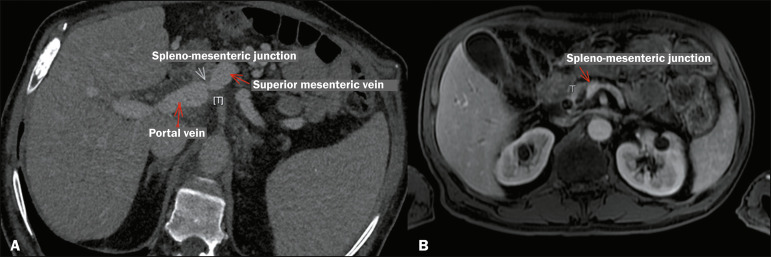
Axial contrast-enhanced CT scan of the abdomen (A), in the portal phase, and axial magnetic resonance imaging scan of the abdomen (B), in 3D T1-weighted sequence, showing a tumor (T) in the head of the pancreas in contact (< 180°) with the spleno-mesenteric junction in a 67-year-old woman. Note the focal tapering and irregular contours of the tumor. Borderline-resectable tumor. IRE was performed intraoperatively as an adjuvant procedure.

**Figure 8:**
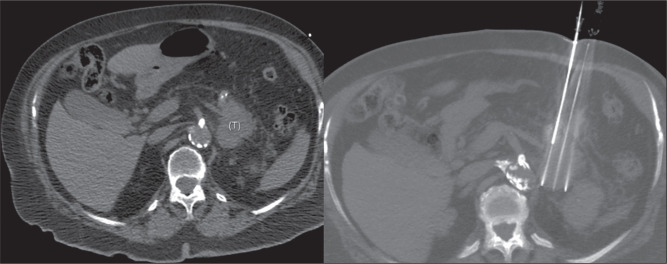
Unenhanced CT scan of the abdomen, showing an expansile tumor (T) in the tail of the pancreas, involving the splenic artery, as well as occluding the splenic vein and left renal vein, in an 89-year-old woman. Resectable tumor. The patient had an unfavorable performance status, with multiple comorbidities, and surgery was contraindicated due to high surgical risk. As a therapeutic alternative, CT-guided IRE was used as an exclusive procedure, in combination with chemotherapy.

Venous involvement, as illustrated in Figures 7 and 8, has less impact on the definition of resectability than does arterial involvement. In most cases, segmental resection and reconstruction are possible even in the presence of venous thrombosis or pronounced infiltration. In such cases, IRE can increase the chances of successful surgical resection with tumor-free margins.

### Involvement of other structures

**Figure 9:**
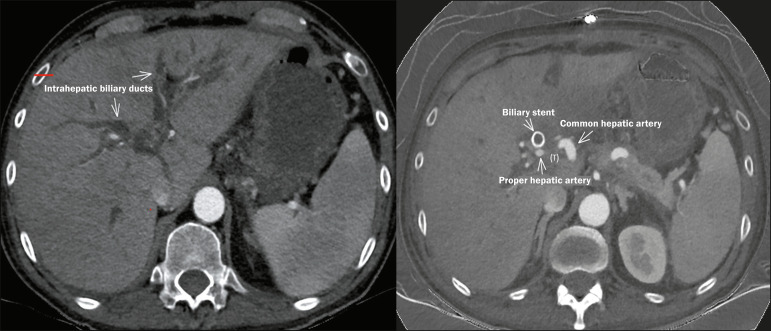
Axial contrast-enhanced CT scans of the abdomen, in the portal phase, showing a tumor, centered in the head and uncinate process of the pancreas, in a 69-year-old man. Note the extensive involvement of local anatomical structures, especially the gastric wall and the third portion of the duodenum. The tumor was also in contact (> 180°) with the superior mesenteric artery and the common hepatic artery. There were also liver metastases. Unresectable tumor. Involvement of hollow viscera walls represents a contraindication to IRE (risk of perforation). In this case, it was possible to perform CT-guided stand-alone IRE based on confirmation by endoscopic ultrasound that the wall infiltration was segmental, with integrity of some layers. Although duodenal or gastric involvement is not one of the criteria of the American Joint Committee on Cancer TNM system, evidence in the literature indicates that it is an isolated factor with an impact on disease survival^(11)^.

**Figure 10:**
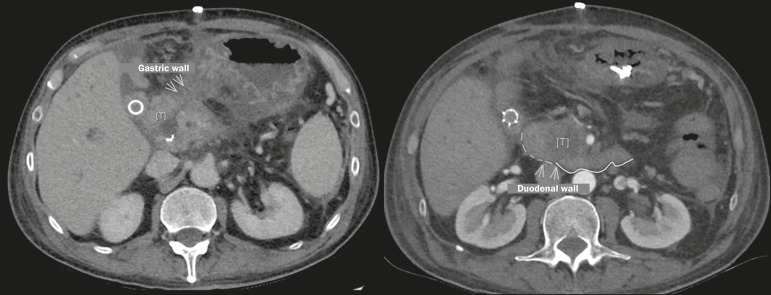
Contrast-enhanced abdominal CT scans, in the axial plane, showing a tumor [T] in the head of the pancreas, in a 79-year-old man, with extensive local involvement, highlighting infiltration of the second portion of the duodenum, the common hepatic artery, and the proper hepatic artery. Endoscopic ultrasound could facilitate the evaluation of infiltration of the layers of the duodenal wall, which represents a contraindication to IRE. Note also the malignant obstruction of the bile ducts, a complication occasionally observed in PDAC and that requires treatment with a biliary stent before IRE can be performed, because of the risk that the edema generated by the procedure will worsen the obstruction.

In tumors with a high risk of infiltration of the walls of hollow viscera (Figures 9 and 10), such as the duodenum and stomach, the indication of IRE must be carefully considered, in view of the risk of rupture. In such situations, evaluation by endoscopic ultrasound is useful for confirming or ruling out the feasibility of IRE. Complications such as malignant obstruction of the bile ducts and stenosis of the portal vein must be treated before IRE can be performed, given the risk of occlusion of those delicate structures by post-treatment edema.

## FINAL CONSIDERATIONS

**Figure 11:**
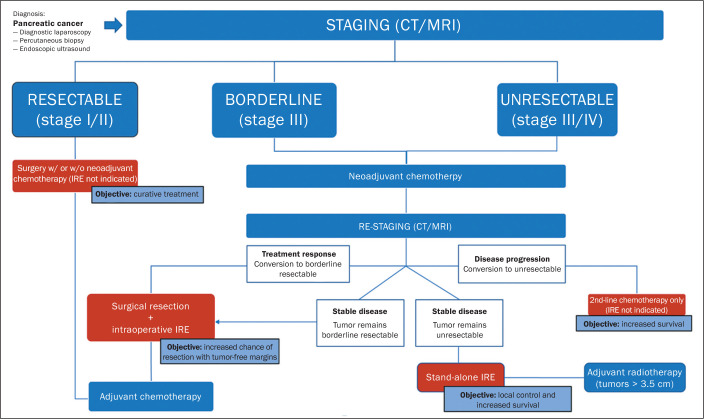
Flow chart of practices in PDAC, highlighting the role of IRE.

Radiologists play a fundamental role in the management of PDAC, not only in the surgical planning but also in the indication of therapeutic alternatives such as IRE. This new treatment modality for locally advanced tumors has shown gains in survival in comparison with the standard treatment of chemoradiotherapy only^(6)^. In certain situations, IRE is an adjunct to conventional surgery, increasing the likelihood of achieving resection with tumor-free margins^(4)^. Due to potential complications, the procedure is considered high risk and should be indicated judiciously. The flow chart shown in Figure 11 systematizes the therapeutic approach in the various stages of the disease.
